# An interview with Rodrigo F. Viecilli

**DOI:** 10.1590/2177-6709.21.5.026-038.int

**Published:** 2016

**Authors:** Renato Parsekian Martins, Marcio Almeida, Armando Yukio Saga, Orlando Tanaka, Sergei Caldas Rabelo

**Affiliations:** » MSc, PhD and post-doc in Orthodontics, UNESP - Araraquara. » Sandwich doctorate, Baylor College of Dentistry, Dallas, TX/EUA. » Assistant Professor of the Post-graduation program in Dental Sciences, Orthodontics, UNESP - Araraquara. » Associate Editor of the journal Revista Clínica de Ortodontia Dental Press.; » MSc in Orthodontics, Bauru School of Dentistry, FOB-USP. » PhD in Orthodontics, Bauru School of Dentistry, FOB-USP. » Post-doc in Orthodontics, Bauru School of Dentistry, FOB-USP. » Head Professor of Masters Program in Orthodontics, North Paraná University (UNOPAR), Campus of Londrina/PR. » Mini-residency in Orthodontics, University of Connecticut/USA. » Author of the book "Ortodontia Clínica e Biomecânica" (Clinical Orthodontics and Biomechanics).; » Professor, Residency in Orthodontics, School of Health and Life Sciences, PUC-PR. » Diplomate by the Brazilian Board of Orthodontics and Facial Orthopedics. » Fellow of the World Federation of Orthodontists (WFO).; » Professor, DDS and Certificate in Orthodontics, School of Health and Life Sciences, PUC-PR. » Pos-doctoral fellowship at Center for Advanced Dental Education, Saint Louis University (St. Louis, MO/USA). » Diplomate by the Brazilian Board of Orthodontics and Facial Orthopedics. » Fellow of the American Association of Orthodontists (AAO). » Fellow of the World Federation of Orthodontists (WFO).; » Adjunct Professor of the discipline of Infant Clinic/Orthodontics, UFRN. » Professor of the Specialization Course in Orthodontics, ABO/RN. » Master and PhD in Orthodontics, UNESP - Araraquara.

**Figure:**
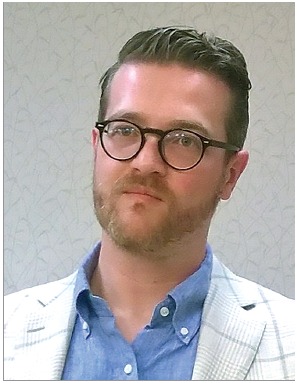

Early in the 2000, digital communication brought me a new friend. We haven't studied together or been presented by any common friends. Along lines and lines of conversation, I met a young orthodontist from Canoas/RS, who even without having studied in one of the traditional Mechanics schools in Brazil, knew it deeply. Over time, I learned his story; he had studied Engineering and, motivated by his parents, graduated in Dentistry and took specialization in Orthodontics. The thirst for knowledge led him to, by his own, contact Dr Charles Burstone, who, by the time, got impressed and invited him to take Masters in Orthodontics and PhD in the USA, being one of the first Brazilian orthodontists with this title from an American university. Besides being awarded with the doctor of philosophy degree, by the Indiana University, he was also the first Brazilian to receive one of the most important award from the *American Association of Orthodontics*, in 2009. For his paper "*Orthodontic mechanotransduction and the role of the P2X7 receptor*", he received the Milo Hellman Award, granted to the best research of the year in the USA. Since then, he has been lecturing almost every year in the *American Association of Orthodontics Annual Session*, presenting 6 lectures in 7 years of congresses. He was professor of Orthodontics in the New York University and, recently, was hired by Loma Linda University, where he teaches full time and is responsible for the Biomechanics lab. He is diplomate by the *American Board of Orthodontics*, and in his free time he cooks and reads about philosophy. It is a great honor for me to have this opportunity to coordinate the interview with one of the great personalities of the Brazilian Orthodontics in the USA: My friend, Dr Rodrigo Viecilli. 

No começo dos anos 2000, a comunicação digital me trouxe um novo amigo. Não tínhamos estudado junto ou sido apresentados por amigos em comum. Ao longo de linhas e mais linhas de conversa, conheci um jovem ortodontista de Canoas/RS que, mesmo sem ter estudado em uma das escolas tradicionais de mecânica do Brasil, a conhecia profundamente. Ao poucos, fiquei sabendo de sua história; que ele havia cursado Engenharia e que, estimulado pelos pais, fez Odontologia e cursou a especialização em Ortodontia. A sede de conhecimento fez com que ele, de maneira independente, começasse a se comunicar com o Dr. Charles Burstone, o qual, ao longo do tempo, se impressionou e o convidou para fazer o mestrado em Ortodontia e o doutorado direto, nos EUA - sendo um dos primeiros ortodontistas brasileiros com esse título por uma universidade americana. Além de ser agraciado com o *doctor of philosophy degree*, pela *Indiana University*, ele também foi o primeiro brasileiro a receber um dos prêmios mais importantes da Associação Americana de Ortodontia, em 2009. Pelo seu trabalho "*Orthodontic mechanotransduction and the role of the P2X7 receptor*", ele recebeu o *Milo Hellman Award*, prêmio concedido ao melhor trabalho de pesquisa do ano feito nos EUA. Desde então, ele tem palestrado quase todos os anos no congresso da Associação Americana de Ortodontia, totalizando 6 palestras em 7 anos de congressos. Ele foi professor de Ortodontia na *New York University* e, mais recentemente, foi contratado pela *Loma Linda University*, onde leciona em tempo integral e é responsável pelo laboratório de Biomecânica. Ele é diplomado pelo *American Board of Orthodontics* e tem como *hobbies* cozinhar e ler sobre filosofia. É com muita honra que tenho a oportunidade de coordenar a entrevista com uma das grandes sumidades da Ortodontia brasileira nos EUA: meu amigo, o Dr. Rodrigo Viecilli. 

Renato Parsekian Martins - coordinator of the interview 

## Why did you choose being professor of Orthodontics in a great American university? What moved you in this decision? (Marcio Almeida and Renato Martins)

While I was studying Engineering, in the mid-90s, my father, Prof. Orlando Viecilli, was specializing in Orthodontics. Frequently, I was questioned about problems in Orthodontics presumed to be from Physics and how to best move the teeth. I found interesting this difficulty in communication - a difference in language for asking and answering questions. I realized that this difference was due to the tradition in Orthodontics language: A certain level of disconnection between physical sciences and the way clinical Orthodontics was traditionally taught. I remember how the word "torque" was used, referring to the inclination of the bracket or to the torsion on the wire. And I wondered how could a person know if the force system released was just a moment, when in fact it is not. The language we use for communicating is extremely important, because the thought depends intrinsically of the language we use to reason. 

I understood, then, that I could somehow contribute to Orthodontics, to help solving this discrepancy. And that, along with other factors, made me change the course of my studies. The greatest challenge was to go through four years of training in which Dentistry traditional teaching is, oftentimes, based on memorization, protocols and techniques, very different from Engineering study. 

At the college in which I studied Dentistry, I had the privilege of being stimulated to be creative, while student, by the professor Oppermann (Department of Periodontics of the Federal University of Rio Grande do Sul), who obtained his doctorate abroad and talked about Periodontics in a very logical way, connected with basic sciences. He explained the molecular mechanisms and cellular interaction in the periodontal ligament during infection, in order to explain what would exactly occur clinically. He based clinical practice with scientific findings, which he would mention during class. He was the only one at college who addressed the specialty like this in the end of the 90s. Ironically, he was the first periodontist who, while I was a student, gave some consideration to the application of advanced mathematics in Dentistry (regarding an idea that I had, at that time, for a periodontal index, which ended up being too complicated to become practical).

At the same time, I studied traditional Orthodontics with my father, Dr. Orlando Viecilli, and with Dr. Armando Hiraoka, during college, and I observed that there was a lot to be clarified in Biomechanics. After I learned the basic of Orthodontics and the treatment according to Tweed and Ricketts techniques, I realized there were a lot of instructions on "how to", but few explanations on the reasoning to do them - and, when these existed, they made no physical or mathematical sense for me, or they had not enough proofs or data to support or refute the instructions, which was extremely confusing.

While I read books of several authors, trying to find explanations, I noticed that most of them were based in opinions, not in experimental findings. In the academic environment of the Brazilian Orthodontics, I noticed some traditionalism, which did not favor creative thinking, and the only way out of this trap was the library. I decided to organize in chronological order the papers I found, so I could understand how Biomechanics evolved in Orthodontics. Back then, it was not this easy to find papers online like it is now. 

Only when I read the papers of Professor Charles Burstone and his colleagues that I found adequate physics explanations for the mechanical problems. In 2001, I translated one of his books, for my own study, and I found some problems in the mechanical design of the "T" loops and how the tests were conducted. At the same time, Professor Mauricio Sakima was publishing papers describing segmented appliances, with logical biomechanical systems, and we invited him to visit Rio Grande do Sul state (Brazil) and give a lecture. Around 2001, I sent Burstone an email with several questions, and, after starting a discussion, he suggested me to publish the book in Portuguese. The numerous discussions derived from these questions, this translation and my general interest in Biomechanics resulted in the first paper I wrote, being a student of specialization in Orthodontics in Brazil, published at the *American Journal of Orthodontics* *^1^* *.* Prof. Burstone was the mentor of my academic career since then and, in a discussion we had about mechanotransduction (conversion of mechanical stimulus into cellular activity), he suggested me to go to Indianapolis for a PhD with one of his former students, focused on the interface between bone biology (*Indiana University,* with W.E. Roberts, highest authority in Orthodontics in bone biology) and Mechanical Engineering (T. Katona and J. Chen, engineers of the *Purdue University,* which graduated many NASA astronaut engineers). According to Prof. Burstone, at that time, that was the only university specifically researching the biological response to mechanical stimulus, with the rigor of Engineering - the way I was looking for. Prof. Burstone and I kept friends and have worked together many times, since then.

When I finished my PhD, it was natural to follow my line of research, and I was invited to initiate the academic career as a professor. Prof. Burstone used to say that the good side of the force (Scientific Biomechanics) needed another "Jedi" to fight against the dark side (the gurus and prophets of orthodontic techniques). Idealistically, I see my career, of scientist and orthodontist, as a continuation of that vision. After that, I did not have much contact with the orthodontic environment in Brazil, and my scientific presentations have been focused on USA and other countries. But I'm glad that there are clinicians in Brazil being trained alike. Brazilian Orthodontics owes much of it to UNESP-Araraquara, to Professors Tatsuko and Mauricio Sakima, Luiz Gandini and Renato Martins. They propagated this way of thinking. 

## Reading and knowing your papers, I realized your close relationship with Dr. Charles Burstone, as your mentor for the scientific study of Biomechanics. In your opinion, how was Orthodontics influenced by him, the greatest researcher of Biomechanics in the world? Why do we need to understand Biomechanics in order to treat our patients? And what course Biomechanics will follow in the near future? (Marcio Almeida) 

In my opinion, there are only a few really brilliant and visionary minds in the history of Orthodontics, from the scientific point of view. Choosing two of them to mention, I need to say Calvin Case and Charles Burstone. As I said before, I always had historical interest in understanding how the thinking has changed through time in Orthodontics. Early in the twentieth century, contrary to all the others, Calvin Case used to defend extractions, in selected cases, in order to obtain a harmonious profile; and controlled tooth movement, with custom appliances, to achieve these objectives. In his book, he described his first scientific efforts to understand how to control a force system and move the teeth with a primitive concept of center of resistance. Case defended specific treatment objectives based on esthetics. On the other hand, Angle, who became far more famous, designed and taught how to use the appliances pre-manufactured by S.S. White; he classified the malocclusions to promote the treatment protocols with his appliances; and was against extractions, with the argument of resemblance between men and divinity. These discussions are available for reading in the old publication *Dental Cosmos* and, in my opinion, it should be read by every orthodontist - for they still happen nowadays, only in different suits. Oftentimes, I joke saying that I'd rather be part of the Case's Society than the Angle's one. Calvin Case was much ahead of his time, but, unfortunately, he was rejected by the orthodontic community. He couldn't take this rejection and ended up committing suicide. Angle and the Orthodontics with Edgewise appliances had the marketing support of a great orthodontic company, unlike Case's ideas. 

Once, I was surprised when discussing with professor Burstone about this subject and the difficulty to convince part of the orthodontic community to accept a more scientific approach to mechanics, instead of focusing on brackets and gurus supported by large companies and convenient protocols. He told me that, not only he had read Calvin Case, but Case had been his greatest influence in Orthodontics. He said that the occlusogram idea, and the efforts to define the center of resistance and movement of tooth came out of the Case's book reading along with the discussions with Prof. James Baldwin, from *Indiana University.* The most interesting is that Prof Burstone had the scientific and political intelligence, charisma and, above all, humility to leave the pedestal of orthodontic tradition and search for engineers and other scientists to help defining the scientific basis of our specialty. Differently from Case, Burstone achieved academic success formulating much of the scientific basis of the orthodontic practice in our clinical daily practice, because he was both a political and scientific master. 

The scientific basis of Orthodontics is to comprehend how to move a tooth in a predictable way, not how to place or manufacture appliances. Literature already agrees that dentoalveolar changes are the biggest ones we achieve in treatment, and that we mold bones and teeth when we intend to take them from position A to B. Teeth with more bone support guide the movement of teeth with less bone support and need to be treated with a consistent force system from A to B, before placing a continuous archwire to serve as a basis for the shape of the arch. In order to obtain the best movement from A to B, we need to know the force system and its effect on the periodontal ligament and bone remodeling. To optimize the system, we need to know materials and biological response to them. This is Scientific Biomechanics. Shouldn't we have a professor with high level of knowledge on Biomechanics in all post-graduation programs? Shouldn't an Orthodontics program be defined by this, instead of by which treatment "philosophy" it follows? 

Craniofacial growing and the diagnosing tools we better emphasize and study during our training are at stake, but Lysle Johnston and Sheldon Baumrind have already shown that we never know, exactly, how much a patient will grow. What is the point of a high refinement in diagnostic concepts and treatment planning if there is no understanding and training on how to properly move teeth from A to B? I could be ambitious and say that, if there is any growth control and treatment objectives we so much like achieving, these will only be achieved with Scientific Biomechanics. Also, knowing Biomechanics allows understanding which are the realistic treatment objectives, regardless of the appliance we use, because all the appliances can be "reduced" to a force system and anchorage that you already know or used before.

It is always good to emphasize that the orthodontic technique or the bracket type to be used is not Scientific Biomechanics. I would like to imagine that the future clinical Orthodontics will completely quit using this language and inaccurate techniques, and will stop highlighting the latest bracket, treatment protocol or appliance. I see a tendency in this direction. In American scientific meetings, for example, generally, orthodontists sponsored by companies with conflict of interest are limited to speak in the commercial area. This needs to be changed in Latin America and other places of the world. More science, less commercialism. I get sad when I see people misusing the word Biomechanics. For example, I have been in lectures of several professors with the title "*Biomechanics of self-ligating brackets*". I have already seen good biomechanical analysis, but sometimes people give a basically commercial lecture about self-ligating brackets and show a number of selected cases and clinical studies with positive results, to convince the audience to use such bracket. I think there is nothing wrong in promoting a product, but it would be good to convince people because it is superior, firstly from the basic sciences point of view and, then, with clinical studies confirming this with a systematic analysis. Only a few do this. 

The greatest challenge of Scientific Biomechanics in Orthodontics is that many choose Dentistry to get rid of Mathematics and Physics and, during training, also from critical thinking. I think it is important to be interested in Philosophy of Science and Language, to understand what really affects our opinion and how to properly support an argument or convince my students not to choose shortcuts to better practice Orthodontics. Every group of post-graduation students I have, even before I start teaching them Biomechanics, goes through 8 hours of examples and counterexamples of what is a scientific and pseudoscientific thinking in Orthodontics, and about how to detect a argumentative fallacy. I think this logical and philosophical perspective is necessary to open our minds to science and creativity in research and treatment.

## In a paper of yours, published at AJODO in 2013, you present a new concept of axes of resistance**^2^** . How can this new concept clinically affect the design of mechanics where it is necessary a movement of intrusion of the anterior block or molars distalization by translation? (Marcio Almeida) 

During my PhD, while performing many finite element analyses, to understand the movement of a tooth, I realized that there was no perfect reference for translation in all directions. How come no researchers were talking about it? Maybe they thought they were doing something wrong. So, in this paper you mentioned, I decided to clarify the possible error range, due to methods, and proved that these differences in references were not a mistake. Actually, there is not a center of resistance, but no one had ever the courage to say that! First, in a rigorous paper I wrote to AJODO^3^, when I was a PhD student, I was already convinced of that, but I just subtly mentioned, because I still had not gathered enough data to prove such a thing. 

Dr. Burstone and I discussed about it and he agreed with me when he saw the data. Then, we elaborated a way to change the concept, based on the obtained data. Based on those discussions, I wrote a chapter, in the last book published by Prof. Burstone, explaining the evolution of the center of resistance concept and why, in fact, it doesn't exist. The original concept is focused on teeth with ideally symmetrical root shapes - for example, a paraboloid of revolution. When the root is perfectly symmetric, the reference for translation is the same in all directions. It doesn't occur with real teeth or group of teeth, because the ligament strain patterns are different in each direction. The objective of this paper was to formalize a scientific definition of a physically rigorous referential for tooth movement. As scientist and orthodontist, I was embarrassed about the scientific basis of tooth movement not having a more significant Mathematics basis, and decided to dedicate some time to fix this. Since then, formal orthodontic Biomechanics study groups^4^ already accepted the fact and confirmed the center of resistance being a myth.

The clinical relevance of this finding is that I estimate that may be a variation of up to 1-2 mm in the translation referential, depending on the force direction. The more asymmetric, the bigger variation. It seems little, but 2 mm may be the difference between a controlled inclination and a translation! I don't have specific information about the scenarios you have asked, but my recommendation for any treatment with Scientific Biomechanics is: Always use clinical feedback, observe how the teeth moved and compare; photograph. If the tooth did not move as planned, make critical adjustments on the force system. Ask yourself: Why it did not work? Answer it in details, with diagrams, and try to understand what is happening. Do not rely purely in ideal M:F ratios, because bone anatomy and variations in brackets geometry, along with deformation potential of the appliance - by structural stress relief and deformation of the appliance by the patient (maybe the most common cause) - may cause deviations from the correct trajectory.

## In a recent paper of yours, published at AJODO, are represented the effects of two types of skeletal anchorage mechanics**^5^** . These mechanics have been advocated by Dr. Chris Chang for the distalization of the entire lower dentition, on the treatment of skeletal Class III, and correction of Class II, instead of using miniplates. What is your opinion regarding the use of this type of anchorage you have been studying by means of finite elements? Do you think about it as the future of skeletal anchorage (mini-implants)? (Marcio Almeida)

The analysis to predict tooth movement that I have been doing for Professors Roberts and Chang are based on the hypothesis that the initial pattern of tooth movement, due to periodontal ligament strain, remains basically the same with time. This is reasonable in inclination movements because the cellular response of the periodontal ligament follows pretty much the compression pattern determined by the main stress set in the beginning, and it changes only a little after the remodeling of the alveolar process. I showed this in another study of mine^6^. This concept was experimentally demonstrated in animals for the first time in this paper which, basically, shows that, from the cellular point of view, a tooth moves according to the pattern of forces and moments, and the consequent effect on periodontal ligament. We presume it is true, for humans, when we place an appliance based on the M:F ratio established for the tooth movement. But do you believe that this was never histologically proved until this paper was published? Similarly, resorption response follows a similar pattern, but it is exacerbated by the ligament necrosis. Establishing a scientific basis for the orthodontic treatment is to give scientific credibility to our specialty.

Surprisingly, I faced a lot of resistance to this demonstration, because it increases our level of responsibility. Many still prefer to believe that orthodontic movement and root resorptions are almost unpredictable, due to biological variations and in the bone resorption pattern. This is true in some cases, but maybe not in the majority. For this reason, I decided to show that the initial ligament strain, predicted by the finite elements methods, is very useful to predict tooth movement in the future and also works in clinical cases. This motivated me to help proving it with Prof. Chang's implant cases, which are very interesting. This clinical reports show that we can predict the movement in a reasonable way using biomechanical principles.

## What precautions would you recommend when using statically indeterminate systems in Orthodontics? (Sergei Caldas) 

In the previous answer I made some comments that can be applied to the errors in general force systems. I think that indeterminate systems are, usually, more complicate to adjust, because they need a measuring instrument, as a transducer, which by itself is in error - due to the absence of periodontal ligament in the measuring process. When clinically adjusting an indeterminate system, such as a loop or a transpalatal arch, only a few orthodontists have enough knowledge to identify, qualitatively and accurately, what is happening. So, my recommendation when using statically indeterminate systems is: Before clinically using them, study hard and try to comprehend the qualitative consequences of making bends or activations. Indeterminate systems offer greater difficulty because it is necessary to accept some uncertainty on the magnitude of forces and moments. My first recommendation for my students is to use the system they know better, from the mechanical point of view. For example, in some cases of symmetrical anchorage, it is possible to make a sliding mechanics to work so well in closing spaces than a "T" loop correctly activated. But in other cases, in which the arch does not slide so well, due to inclination discrepancies between teeth, a "T" loop and a frictionless mechanics may be the best option. I am against the use of a specific method for all cases: There is always a simpler and more efficient treatment, according to the scientific circumstances of the case. In my opinion, there is no honor in using complicated systems in all the cases. Maybe there is greater intellectual importance in being wise to choose the best system for the case you have in hands at the moment. 

## The use of cantilevers for verticalization of molars is a biomechanical resource commonly used by orthodontists. However, many authors claim that increasing the lever arm, using the same vertical activation, would increase the moment produced for the molar verticalization. Do you agree with this? And which is, effectively, the moment responsible for promoting the molar movement (moment of the force or moment of the binary within the slot)? (Sergei Caldas)

There are many questions at stake here, and I think that the problem originating this controversy between authors is in understanding the concepts of: Moment, load/deflection ratio, balance (first law of Newton) and equivalence of force system, allied to the problem of inaccurate language in Orthodontics, to which I have referred before. In this case, the problem of language is the word "activation"; it is a confusing word. The more accurate words to use would be maximum deflection of the cantilever and force released by the cantilever.

**Figure 1:**
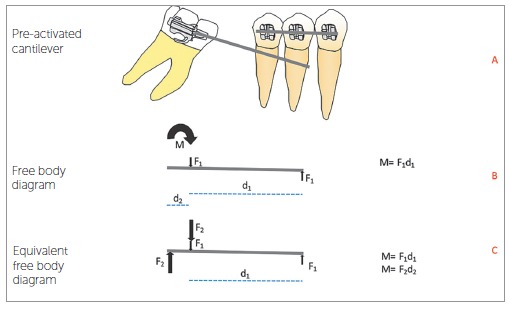
Free body diagram showing the forces on the cantilever wire.

**Figure 2:**
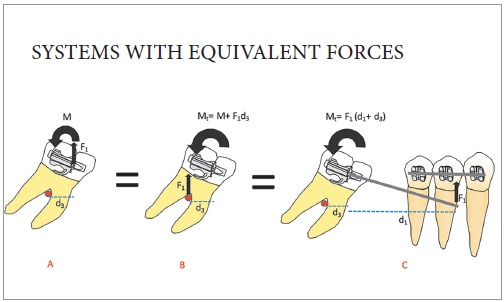
Systems with equivalent forces on the molar, considering it: Isolated, in the tube (A) or in the center of resistance (B); or considering the molar and the cantilever as the same body (C). In the last case, it is not necessary - and normally would be incorrect - to design a curved arrow of the moment on the molar, which was inserted here with a didactic purpose.

The origin of the language problems is that, oftentimes, in Scientific Biomechanics didactics, classes are focused on the appliances. After teaching Biomechanics in the last 10 years and trying to find the balance between rigor and best didactics, I believe the best way is organizing classes by the principles and concepts from Physics and Mathematics. Each concept can be clarified with examples of treatment objectives, force systems and, only then, appliances. Teaching this was, however, requires a long program and logical sequence, and this is not possible in short programs where orthodontists search, for example, for a new "appliance" for molar uprighting. So, to answer your question, I would like to clarify the following aphorisms:

1. In Statistics, the first law of Newton implies that an object at rest or in constant speed has the sum of forces and moments equals to zero. Observe that, to verify a balanced force system, normally we would separate the cantilever in question (as represented by Fig 1A), in what we know as "free body diagram" (Figs 1B and ^1^C) and we would design the force system acting on the wire, as indicated below. Observe that the moment of torque M, represented by the curved arrow, in fact is the pair of forces F2, illustrated in Figure 1C. This is what happens "in reality". The representation of the moment with a curved arrow is purely didactic.
2. After obtaining the force system that the tube applies on the wire, we invert it to obtain the system the wire applies on the tube, and calculate the force system on the molar using the concept of equivalence of force system. Since the forces on the tube generate moments on the molar, the force system in the resulting center of resistance needs to reflect this (Figs 2A and ^2^B).
3. The effect of the total force system on the tube is the same of the equivalent force system on the center of resistance of the molar. The molar is balanced by the first law of Newton, by means of interaction with the dentoalveolar complex.
4. The moment applied to the molar for its verticalization depends on the force measured at the anterior application point, and on the distance to the resistance axis of the tooth orthogonally to the line of action of the force. This is the "fast" way to verify the moment on the molar, if we imagine that tooth and appliance are basically the same "rigid object" and that we are interested only in what happens with the tooth (Fig 2 C).
5. The increase in length of the lever arm (distance) increases the moment on the molar, if the force measured is kept constant.
6. In a cantilever of linear material, such as steel or beta-titanium, the load/deflection rate is inversely proportional to the cube of the distance - that is, by bending the lever arm, there is a reduction of 8x if the deflection is constant; that is, doubling the distance, we would need to increase the deflection 8x to obtain the same force and double the moment on the molar.
7. The lack of knowledge of any of the concepts aforementioned, along with the misuse of the word activation (confusing force and deformation), is what causes the problems you mentioned.

**Figure 3:**
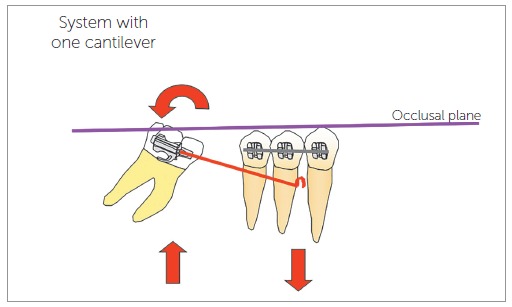
Simple cantilever system showing the forces and moments in the posterior and anterior elements (pseudodiagram of free body). The load/deflection rate is inversely proportional to the cube of the distance of the tube to the hook (lever arm).

Ideally, a person who does not understand the basic principles of Statics, taught in high school, should not teach Biomechanics and we should not have this kind of controversy in our profession. On the other hand, from the positive point of view, when someone publicly teaches something wrong, frequently due to the lack of a formal Physics basis, there is at least the chance to discuss it. In the end, I like to believe that the search for knowledge of fundamental basic sciences always happens. 

## Last year, Orthodontics lost one of its greatest scholars of Scientific Biomechanics, Prof. Charles Burstone. Having lived directly with him, and being one of the greatest researchers of his work, how do you see the legacy he left in our specialty? And, also, what do you consider being fundamental and that every clinician should know of his work, to practice an Orthodontics of excellence? (Sergei Caldas)

The difference between Burstone and other great names in Orthodontics is that he did not teach a technique, he was not a guru or a clinician. He was a scientist. Every method or system he analyzed in Orthodontics was evaluated from the scientific point of view, not from the empirical point of view or from "what works in my clinic" or "in my hands". Comparing the legacy of Prof. Burstone with any other left by an orthodontist who claimed a technique based only in his clinical reputation is not fair. I think that every scientist of Biomechanics should make it clear. Many around him, even students, wanted to name his mechanics "segmented arch technique", something that I think is a huge mistake. For example, when a "T" loop is recommended, instead of any other loop, it is because the entire Biomechanics science defends that this is the best simplified design of a loop to obtain the minimal load/deflection rate and M:F ratio.

In my opinion, the legacy of Prof. Burstone is simply the institution of the science in orthodontic mechanics. One great reason for me to keep teaching and researching is that I feel honored in preserving this legacy. My relationship and long weekly conversations with Prof. Burstone, and the great friendship and mutual respect we had in our discussions are a great motivation. I feel extremely privileged for having had the opportunity of questioning and learning pretty much everything he had to teach, and I am responsible, as a scientist, for keeping his legacy.

## The word Scientific Biomechanics is still little known by Brazilian orthodontists. What does this word really mean, and how its use can help clinicians in a more rational approach of their treatment plans? (Sergei Caldas)

Biomechanics is based on observation of facts and experiments, and it keeps an open mind for the changes in guidelines, depending on what is observed. It is not about "helping" the clinician, but about learning the correct way of doing things, with a scientific basis. Burstone once told me that there was never such a thing as "Burstone's segmented arch technique". The appliances always changed according to the materials and studies on M/F and on how to adequately move the teeth. People started referring to these procedures as a technique because other procedures had also been called techniques. It is not fair to compare pure science with technique. The way the procedures have evolved in Scientific Biomechanics is documented in literature, with scientific reasoning and proofs since the 50s! What "technique" can do the same?

Traditional orthodontic techniques are based on protocols and prescriptions acceptance because they are supported by an assumed expert or by the marketing of a bracket or appliance, and not in scientific explanations. For this reason, frequently the argumentation justifying techniques is based on logical fallacies and a bias of selected quotations of clinical studies. Learning to identify fallacies is important so that you won't get carried by commercialism, especially in Brazil. Knowing the Scientific Biomechanics possibilities for a case releases the orthodontist from techniques, and forces him to diagnose and plan a more specific treatment, at least as a mental VTO. The use of Scientific Biomechanics allows, as a matter of fact, to scientifically reach the objectives of this VTO.

## How does the study and knowledge of Biomechanics in Orthodontics affect the diagnosing and mechanics planning of clinical cases? And how, from the scientific point of view, is Biomechanics related to contemporary techniques in Orthodontics? Orlando Tanaka

I think that almost every orthodontic technique has something that, coincidentally, can be used and improved from the scientific point of view. For example, the technique of closing spaces with sliding mechanics can be very easy and effective, for cases without great anchorage challenges, if the clinician is concerned on using the most possible rigid archwire and verifying if the wire slides easily in the anteroposterior direction. It is clear and common sense from the mechanical point of view, but in my experience it is not common to find a person trying this before setting up for space closure. When the wire is not rigid and a curve is formed on the arch, many orthodontists still insist on increasing the force and trying to close spaces, when the friction created by the normal forces at the brackets to maintain teeth vertical is immense. Biomechanical principles are universal and apply to the optimization of simpler "techniques" in Orthodontics. The scientific biomechanic sees a procedure as a series of physical variables, and not as a technique. 

Another tip to decrease friction is that, when I teach my students to use sliding mechanics I ask them: To remove any appliance interfering in the shape of the arch in the final stages of aligning, such as transpalatal or lingual arches; to use the most possible rigid archwire (tempered chrome-cobalt steel), and pull and replace the wire at the brackets, to feel the friction level. If this is not possible, a 200cN force won't have much effect. If the archwire does not slide, it can be beneficial to align with a larger sized archwire to eliminate the normal forces derived from moments in all directions and, then, go back to a smaller sized archwire, trying again the sliding.

Knowing Biomechanics and basic sciences opens the mind for creativity and optimization of technical principles which, *a priori* and by definition, didn't have space for it. This shouldn't be nothing new. Any serious science works this way.

## What are the available tools to improve the knowledge in orthodontic Biomechanics? Are computational simulations essential for this? (Orlando Tanaka)

I think the tools are not the best way to improve research in orthodontic Biomechanics, but the education and knowledge of the fundamental variables of Mathematical sciences ruling Biomechanics and its effects on treatment. 

For example, let us say that a study compares the effects of extraoral and cervical traction appliances for correction of Class II. The authors do not give relevant details on how the appliances were assembled, more specifically which was the action line of the force in relation to teeth resistance references (if the molars are loose from the archwire). There is no explanation and the only difference is that an appliance was used in some of the cases, and another in the other cases. 

What is the utility of the conclusions of papers like this, if the real "medication" of Orthodontics is the force system, not the appliance itself? This is a serious language problem we need to fix in our profession. In Materials and Methods, we need to give every detail about the force system. Studies based only on "appliances" are, oftentimes, almost useless, because the conclusions are limited to a variation of configuration in patients, which is unique to the study itself. There is another problem in study designs: Using a laser instead of a chainsaw to cut a tree. I revise probably the most part of the papers on finite elements published at AJODO. Often, the problem is modeled by placing an entire statically determined appliance, when only the force system applied would be enough, and there is no need to use finite element analysis to solve the problem. This makes me sad. The finite elements method serves to find answers to problems that we can not predict with traditional ways. Science evolves through testing and rejecting hypotheses. The finite elements method helps to reject hypotheses that can not be rejected without the basic application of static principles, or without the modeling of a periodontal ligament in real experiments; or when appliances are too complex to be tested in mass. The choice of a tool is based on what is necessary to test a hypothesis in a simple way. 

## At the AAO Congress in San Francisco/CA, 2015, you presented the SmartArch. What are the main biomechanic advantages of SmartArch compared to more traditional NiTi orthodontic archwhires? What is the relevance of Dr. Burstone in developing the SmartArch project? (Armando Saga and Orlando Tanaka)

SmartArch is an aligning archwire with CuNiTi stiffness requirements calibrated for each interbrackets distance, for cases with or without premolars extraction. It was conceptualized in discussions I had with Dr Burstone about how to design the best possible aligning archwire. However, I am the only responsible for the calculation and development methods, which is intellectually protected.

Basically, SmartArch was developed to be the only aligning archwire in first and second order, for a case where a continuous archwire can be inserted. It is a round 0.016-in or 0.018-in CuNiTi wire. In each interbrackets distance, the wire is treated with laser in order to have a different stiffness. The stiffness modification is made by the selected vaporization of nickel atoms, with laser. 

The stiffness in each interbrackets distance was calculated so that each tooth has an optimized force to be moved. At last, SmartArch is the first wire in Orthodontics considering both the root support and interbrackets distance, and has experimental and finite elements data proving that all the teeth will be submitted to forces with ideal proportions to move them. We also considered the friction, and choosing the wire and recommending the use of metal ligatures or self-ligating brackets is based on these data. Our data show that the use of elastic ligatures is not the most efficient method for alignment in severe orthodontic cases. Self-ligating brackets or with a metal ligature slightly loose have basically the same effect. It occurs because, when a tooth is aligned, the excess of wire needs to slide to posterior. If there is too much friction, the alignment will not be efficient.

## What are your recommendations to improve the graduation of orthodontists? (Orlando Tanaka)

I think the training in Scientific Biomechanics should represent 50% of theoretical classes in Orthodontics programs. 

## What is the reliability of the numerical simulation methods, when applied to the study of orthodontic Biomechanics? (Armando Saga)

If the problem is properly modeled, the error for a mesh typically acceptable proposed by a software will be around 10% for a problem of wires or beams simulation, for example. This can be improved with mesh refinement and taken to less than 2%. The error can be higher if the materials are not properly modeled, of course. By properly, I mean that there must be experimental results supporting the property of the materials. Complex non linear materials, such as superelastic wires, have mathematical models based on approximations which also incur in error. These errors are small and can not be a reason to simply discard the results. Ultimately, the total error depends on each problem being modeled, but it needs to be accepted because, in general, the finite elements method is superior in aspects that the experiments are not.

## Von Mises stress analysis is, frequently, used in numerical studies for stress analysis in biological tissues. What is your opinion on this? (Orlando Tanaka and Armando Saga)

Von Mises criteria basically consists in calculating the so called "total or equivalent stress" of distortion of a material undergoing load. It is based on the theory that the energy of total distortion can be compared to the material elastic capacity, to predict if a ductile material will fail (permanently deform). It can be useful in structural analysis - such bridges, buildings, etc - and engineers normally use it. For this reason, in many cases, papers use von Mises in orthodontics due to a problem in communication of purposes between the orthodontist who asked the analysis and the engineer. In some cases, both don't know exactly how/what to analyze. For the periodontal ligament, there is a gradual answer derived from the main stress. It doesn't make any sense to use von Mises, and I can tell you this because I compared myself the histological results with von Mises and main stresses patterns. The von Mises stress does not indicate biological response in Orthodontics. If I want to verify the potential of bone fracture, this might have some utility. But not for modeling of tissues in general, for analysis of tooth movement. I have already explained this many times when revising papers, and each time I find less this error, because the authors are starting to understand this.

## There is still an aggressive marketing, from some orthodontic companies, regarding the supposed advantages of self-ligating brackets in shortening the orthodontic treatment time. How do you explain the existing differences between ***in vitro*** researches results, showing less friction of these brackets, and the results of ***in vivo*** researches, showing the absence of difference between these and the conventional brackets? (Renato Martins)

Let us forget for a minute that clinical studies exist. There is nothing mystical about a self-ligating bracket. It is just a metallic door which, if passive, has effect similar to a passive metallic ligature, because in both cases there are no normal forces generating friction between the wire and the bracket, or the wire and the ligature. If the metallic door is active, it has an effect similar to a metallic ligature with a low stress. A metallic ligature with stress adds normal forces, which will add friction. An elastic ligature may have the same stress, but will cause more friction because it has a higher friction coefficient. A self-ligating bracket may be easier to manipulate than a metallic ligature. A ceramic self-ligating bracket is more esthetic. This is everything you need to know about self-ligating brackets for taking scientific and logical decisions about them. 

Due to what I wrote above, I think there might be, physically, an advantage in time of treatment, if compared to the orthodontist who aligns and closes spaces using elastic ligatures. Clinical studies help to analyze its benefits and, in general, they show it is controversial and, possibly, marginal. This benefit can be simulated by the use of metallic ligatures slightly loose. In my general analysis of the problem, I think it is at least questionable if this marginal benefit justifies the exaggerated cost of such brackets. The orthodontic companies take a lot more advantage than the patient and the orthodontist. An orthodontist treating the patient with conventional brackets but with superior knowledge of Biomechanics will certainly treat a case faster than an orthodontist who ignores Scientific Biomechanics and uses self-ligating brackets.

The marketing of some orthodontists saying that by using these brackets the treatment will be superior is even more problematic from the ethical point of view. Normally, if the patients are interested and already heard about the subject, I shortly explain what I wrote above. I never lost a patient for not using metallic self-ligating brackets. Following the logic of my explanation, I use lingual or ceramic self-ligating brackets in adults due to the obvious esthetical benefit and shorter time in chair (in case of the lingual appliance). 

## It seems that, generally, in the USA, the orthodontists like treating some types of Class II with upper extractions, while in Brazil it is tried to treat these same cases with molars retraction. How do you see these two treatments and why do you think there is this difference between Brazil and USA? (Renato Martins)

I think here in the USA I became a far more practical orthodontist and abandoned a little the hero complex that led me to proposing treatments via distalization. Here, in general, we want to solve the problem in the faster and more efficient way possible, with the minimal compromising in the quality of the result. Obviously, when we close extraction spaces there are two groups of teeth moving in the same direction. Let us say that the speed of closing spaces is 1 mm/month (0.5 mm/month in posteriors, and 0.5 mm/month in anteriors) to close a 6 mm space and correct an Class II. It means 6 months to correct a Class II. For correcting a Class II by distalization, we must open the same space with half of the speed (x/2), since only one tooth is moving. It means 6 months to correct a 3 mm molar Class II. Then, to retract the anterior teeth, again with half of the speed (x/2), another 6 months. This means it takes a year to correct a Class II by distalization and 6 months by extraction. I am not against distalization, but the fact is that extraction is a far more efficient way of correction. Observe that I did not even mention the anchorage challenges, complications with appliances or mini-implants failures, additional appliances costs, etc. If you explain all this to a patient, which treatment method do you think he would choose, if the profile result is the same?! Maybe some patient who is more radical against extraction, or that have some chance of losing teeth in the future due to multiple restorations, may find some advantage in a treatment via distalizatoin. But these are few.

## There is a huge fear of orthodontists in causing root resorptions while treating a case. What is the relation between the type and intensity of forces and non-physiological external root resorption? Is there a way of the orthodontist trying to avoid it? (Renato Martins)

There are patients who will suffer resorption regardless of the orthodontic treatment, but these cases are rare and, when it happen, the resorption normally stops after removing the stimulus. Unfortunately, there is no scientific basis for what is considered an ideal stress on the periodontal ligament in human teeth. My research is, so far, the only one showing the stress limit to necrosis and exacerbation of root resorption on the periodontal ligament in rats and mice. I have certainty and experimental basis to say that there is a limit of stress to necrosis and, if we keep crossing this limit during treatment, the root will be resorbed. This is clear on my papers and documented by the relation between the stress on the ligament and cellular response. But, then, why are we doing a relatively good job as orthodontists in avoiding resorptions?

In general, we know some things. We know that, if we use a NiTi 0,014-in superelastic archwire to align a lower crowding in a healthy patient, there will not be significant resorption. We know how much force we need to distalize and close spaces. We developed some clinical perception of what is acceptable force in some cases. The damages can occur when we lose this perception of our "comfort zone". This is nothing like a scientific concept, but I think it is important to mention it, lacking a better one, to show how primitive is the scientific state. Concepts about blood pressure and things like that, to propose ideal forces in Orthodontics, were never proved.

Our group has been working for 12 years in creating evidences of what can be an ideal stress range for the ligament, connecting Engineering and Biology, hoping to change this scenario. In the next years, I hope to be able to obtain this evidence for our "fundamental medication".
